# Sustainable Plant‐Based Electrodes for Electrocatalytic Conversion of Small Molecules: From Biomass to Functional Electrodes

**DOI:** 10.1002/EXP.20250158

**Published:** 2026-05-25

**Authors:** Ying Long, Zhijie Chen, Jiangzhou Xie, Jinliang Zhu, Wei Wei, Yi‐Ming Yan, Bing‐Jie Ni

**Affiliations:** ^1^ Centre For Technology in Water and Wastewater, School of Civil and Environmental Engineering University of Technology Sydney Sydney Australia; ^2^ UNSW Water Research Centre, School of Civil and Environmental Engineering The University New South Wales Sydney Australia; ^3^ School of Mechanical and Manufacturing Engineering University of New South Wales Sydney Australia; ^4^ School of Resources, Environment and Materials, State Key Laboratory of Featured Metal Materials and Life‐cycle Safety For Composite Structures Guangxi University Nanning P. R. China; ^5^ State Key Lab of Organic−Inorganic Composites, Beijing Advanced Innovation Center For Soft Matter Science and Engineering Beijing University of Chemical Technology Beijing P. R. China

**Keywords:** electrocatalysis, environmental sustainability, hierarchical porosity, plant‐derived electrode, small‐molecule conversion

## Abstract

Electrocatalysis has emerged as a cornerstone in advancing energy conversion, storage, and the production of value‐added chemicals. A pivotal determinant of electrocatalytic efficiency lies in the design of electrode materials, underscoring the urgent need for cost‐effective electrodes with robust structural integrity, high electrical conductivity, substantial porosity, and excellent catalytic activity. Plants (e.g., wood and bamboo)‐derived monolithic electrodes have garnered growing interest due to their inherent hierarchical porosity and abundant cellulose content, offering significant promise for diverse electrocatalytic reactions. This review focuses on the synthesis techniques of plant‐based monolithic electrodes, highlighting their structural features and evaluating their impact on electrocatalytic performance. Additionally, applications of plant‐based monolithic electrodes in small‐molecule conversion processes, including water electrolysis, oxygen reduction, carbon dioxide reduction, and nitrogen reduction reactions, are analyzed. The discussion culminates in evaluating the persistent challenges and perspectives in electrode material development. These insights provide a roadmap for designing next‐generation electrochemical devices that combine superior efficiency, stability, and environmental sustainability. By advancing the performance of plant‐based monolithic electrodes, this review lays a robust foundation for developing high‐performance, cost‐effective, and sustainable electrochemical materials and technologies for future applications.

## Graphical abstract

This review summarizes recent progress in plant‐based electrodes for small‐molecule electro‐conversion, focusing on synthesis strategies such as carbonization, hydrothermal processing, electrodeposition, and chemical vapor deposition. It highlights the structural features influencing porosity and conductivity, and discusses their applications in water splitting, oxygen reduction, CO_2_ reduction, and ammonia oxidation, along with key challenges and future perspectives.

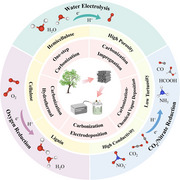

## 1 Introduction

The growing need for sustainable energy solutions has driven research into innovative materials and methods for energy storage and conversion [1]. Electrochemical technologies, including batteries [2], supercapacitors [3, 4], and fuel cells [5], have attracted substantial interest due to their capabilities for efficient energy conversion. Notably, the electrochemical conversion of small molecules, including water, CO_2_, and nitrate, into valuable chemicals and fuels presents significant potential in addressing pressing global energy and environmental challenges [6, 7, 8, 9]. This conversion process is essential for renewable energy storage, carbon capture, and producing ammonia and other high‐value chemicals [10]. Nonetheless, the efficiency and selectivity of these transformations are profoundly influenced by the properties of electrodes within electrochemical cells. The development of electrodes that are not only efficient but also cost‐effective and environmentally sustainable remains a crucial challenge in advancing these technologies [11].

Traditionally, electrodes employed in small‐molecule conversion processes are fabricated from metals such as platinum, nickel, or cobalt, which demonstrate high catalytic efficiency but are accompanied by notable limitations [12, 13]. The lifecycle of these metals from mining and refining to disposal imposes significant environmental and economic costs [14]. Additionally, the rigid and densely packed structures of metal‐based electrodes can impede ion and reactant transport, thereby constraining the overall efficiency of electrochemical reactions [15].

To overcome these challenges, monolithic electrodes derived from natural plants have emerged as a highly promising and sustainable alternative [16, 17, 18]. Plant‐based monolithic electrodes possess a unique, hierarchically porous structure [19], that is naturally optimized for fluid transport, which can be repurposed to enhance ion and reactant diffusion in electrochemical systems [17]. Additionally, plants' high cellulose and hemicellulose content offers a rich carbon source, which can be converted into a conductive and mechanically robust framework through carbonization [20, 21, 22, 23]. This approach aligns with a more sustainable manufacturing philosophy [24], representing a significant shift away from conventional methods [25, 26, 27, 28] that often degrade biomass into powdered carbon, a process that is not only energy‐intensive but also leads to blocked active sites and diminished catalytic performance. By directly functionalizing the intact plant structure, we can create self‐supporting electrocatalysts that exemplify the principles of green chemistry.

**FIGURE 1:**
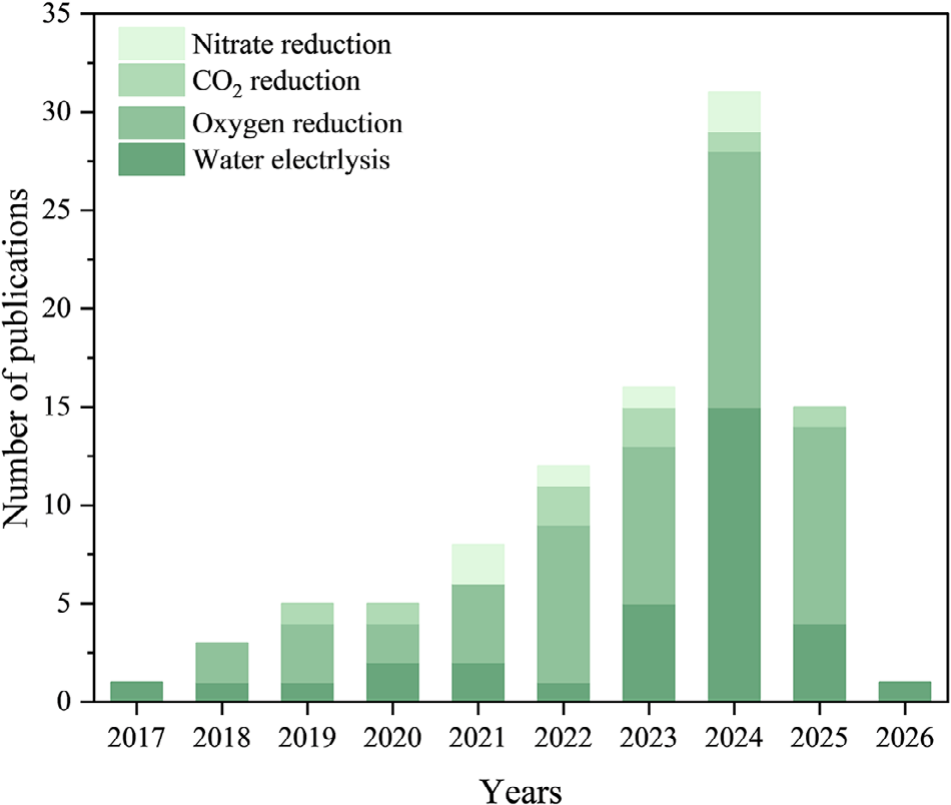
Annual publication output on wood‐based electrodes for small molecular conversion from 2017 to 2026. A combined keyword search was conducted in the Scopus database, for which material‐related keywords such as “wood‐based electrode,” “wood electrode,” and “carbon wood” were combined respectively with keywords for four application areas: “water electrolysis,” “oxygen reduction,” “CO_2_ reduction,” and “nitrate reduction.”

The significant potential of this approach has ignited a surge in research interest. Since 2017, the publication volume of related research has exhibited a significant growth trend, reaching a peak in 2024. This growth was primarily driven by research in water electrolysis and oxygen reduction, which are the two mainstream areas and account for the majority of publications. In contrast, CO_2_ reduction and nitrate reduction are emerging fields with smaller research volumes (Figure 1). Despite this rapid progress, a critical gap persists between laboratory‐scale success and industrial viability. Closing this “lab‐to‐market” gap requires a thorough assessment of how current synthesis methods can be adapted for scalable and cost‐effective production, a perspective that is essential for guiding future [29].

Several reviews have examined the design strategies and synthesis of wood‐based nanomaterials for emerging applications [30, 31, 32, 33, 34]. However, no review has yet comprehensively addressed plant‐based catalysts for small‐molecule electrolysis. This article aims to bridge this gap by summarizing recent advancements in the development of plant‐based electrodes for small molecule transformation. Our analysis places particular emphasis on various synthesis techniques, including one‐step carbonization, carbonization‐hydrothermal processes, carbonization‐electrodeposition, chemical vapor deposition, and electroplating. Additionally, we provide an in‐depth review of the structural characteristics of plant‐based electrodes, particularly regarding their porosity and conductivity. Building on these developments, we then examine the applications of plant‐based electrodes in processes such as water electrolysis (including seawater electrolysis), oxygen reduction, CO_2_ reduction, and ammonia oxidation. Finally, we briefly outline key challenges and potential directions for future advancements in the design and synthesis of plant‐based electrodes aimed at enhancing small molecule electro‐conversion efficiency.

## 2 Synthesis of Plant‐Based Monolithic Electrodes

**FIGURE 2:**
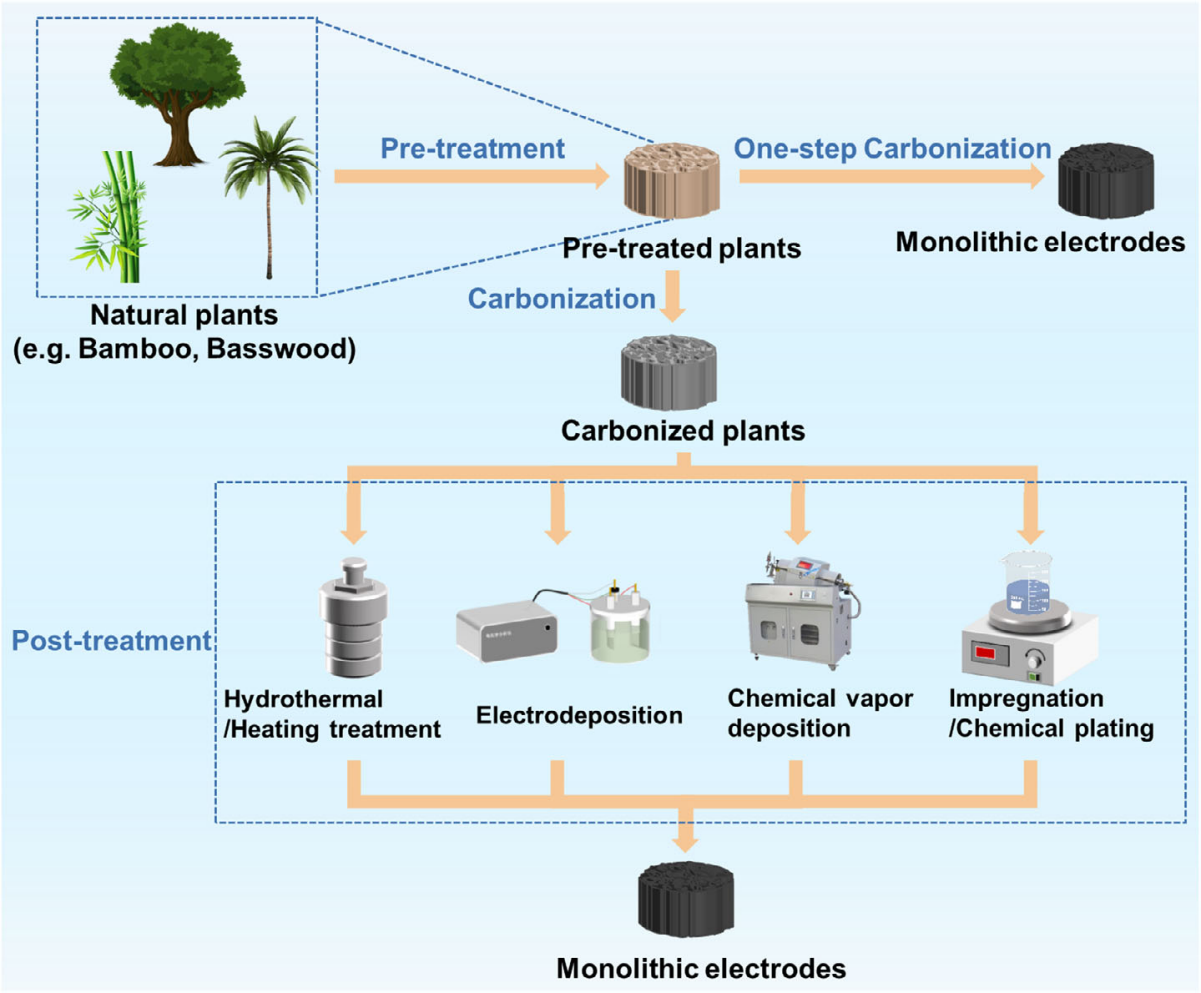
Graphical representation of plant‐based monolithic electrode preparation methods.

To develop a stable, porous structure suitable for use as an electrode material, plants typically undergo processes of carbonization and, in some cases, activation. Conventional synthesis methods for carbonizing and activating natural plants encompass one‐step carbonization in an inert or oxygen‐deficient atmosphere [35], hydrothermal carbonization [36], and chemical activation followed by carbonization (Figure 2) [37].

### 2.1 One‐Step Carbonization

**FIGURE 3:**
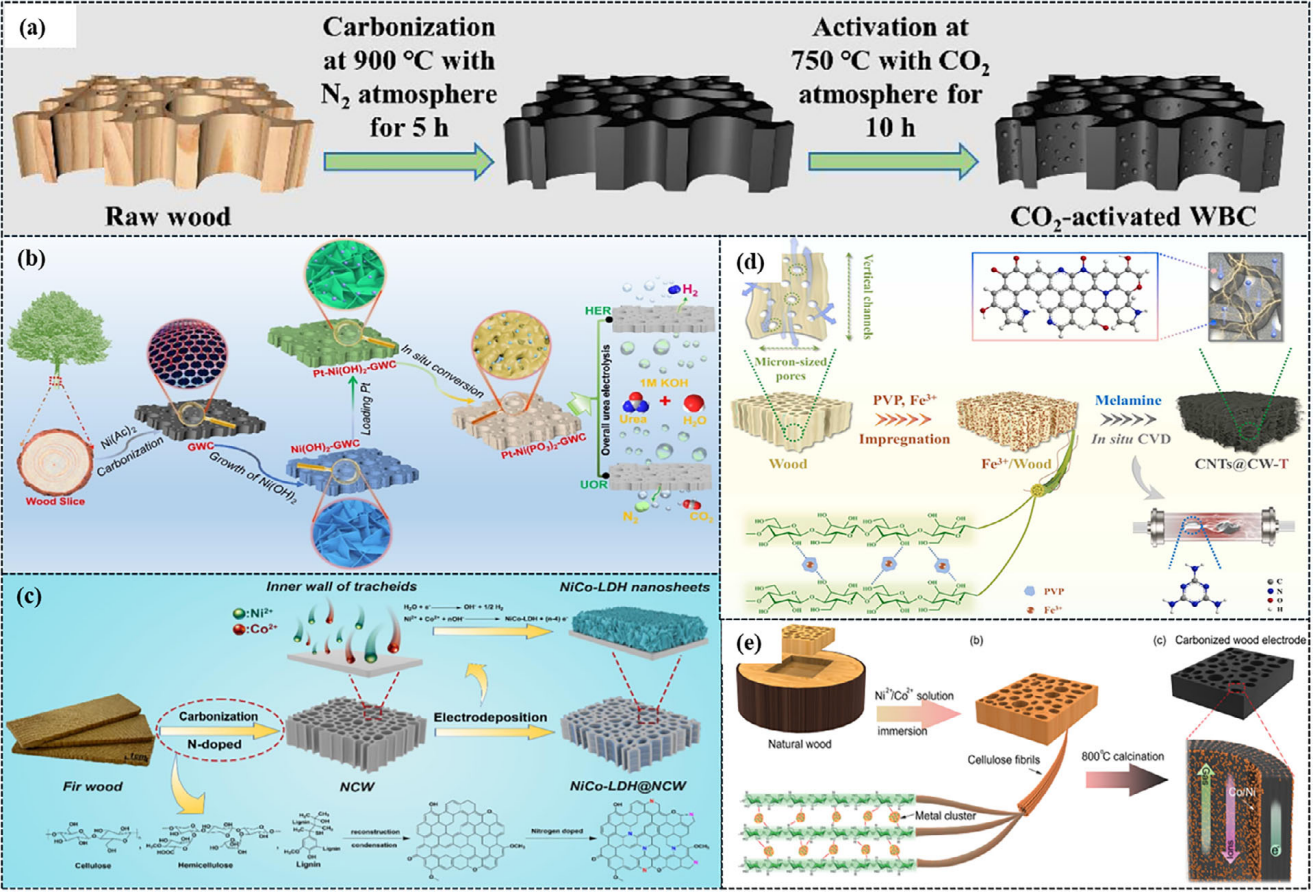
Schematic diagram of the (a) one‐step carbonization. Reproduced with permission [47]. Copyright 2022 Elsevier B.V. (b) Carbonization‐hydrothermal process. Reproduced with permission [48]. Copyright 2024 Elsevier B.V. (c) Carbonization‐electrodeposition process. Reproduced with permission [49]. Copyright 2024 Elsevier B.V. (d) Carbonization‐CVD. Reproduced with permission [50]. Copyright 2023 Elsevier B.V. (e) Carbonization‐impregnation. Reproduced with permission [20]. Copyright 2021 Wiley‐VCH.

One‐step carbonization is a widely utilized technique for preparing plant‐based electrodes, representing the initial and pivotal phase in transforming plants into a conductive material (Figure 3a). This approach involves the direct pyrolysis of biomass sources, such as bamboo and lignin, typically in the presence of a catalyst to yield conductive carbon materials [38, 39]. Notably efficient, this process requires specific high‐temperature conditions for optimal conductivity and graphitization. Lignin‐based precursors may undergo sulfonation, which enhances the catalytic properties of the resulting carbon structures [40, 41]. High‐temperature treatment converts organic components of plants into carbonized material, preserving their intrinsic architecture. Furthermore, it is widely recognized that the hierarchical porous structure inherent to plants can maintain substantial conductivity following calcination in an inert atmosphere [31]. Initially, a suitable plant type (e.g., pine, poplar) is selected, shaped to the desired dimensions, and subjected to a drying process to remove moisture [42, 43]. The pretreated plants are subsequently placed in an inert atmosphere (e.g., N_2_ or Ar) and thermally treated. This thermal decomposition process breaks down cellulose, hemicellulose, and lignin within the plants, ultimately producing a porous carbon structure characterized by a high specific surface area [44, 45]. Li et al. [46] reported on a charcoal electrode fabricated by drying pine plant blocks in an electric oven, followed by carbonization in a tube furnace under an argon atmosphere. This electrode demonstrated notable efficacy in nitrate reduction, highlighting the utility of the carbonization method for producing high‐performance electrodes. Furthermore, the study investigated the influence of carbonization temperature on electrode performance, finding that temperatures between 700 and 900°C had minimal impact on the efficacy of the charcoal anode. However, at temperatures below 600°C, the electrode's conductivity decreased substantially.

One‐step carbonization methods present several notable advantages, such as shortened processing time, cost efficiency, and the capacity to repurpose waste biomass, which contributes to environmental sustainability [51]. Although these methods offer a promising and sustainable approach for producing plant‐based electrodes, the high‐temperature carbonization of plants in an inert gas atmosphere, while straightforward and effective, often results in carbonized wood (CW) with limited electrochemical performance [31]. Consequently, challenges persist in optimizing carbonization conditions to achieve target material properties and in scaling up production for industrial applications. To address these challenges, surface modification of carbonized plants is essential for better understanding and refining the process across diverse electrocatalytic applications. This effort includes the exploration of various biomass sources and catalytic agents to enhance the functional capabilities of the resulting carbon materials. Additionally, post‐carbonization chemical activation treatments, such as the application of activators, are employed to increase specific surface area and porosity [52]. Pure carbon materials inherently offer a limited number of catalytic active sites; however, heteroatom doping is posited to cause charge redistribution among carbon atoms, thereby enhancing the activation of small molecules [53]. Consequently, heteroatom doping has emerged as a pivotal strategy for enhancing the catalytic efficiency of carbon‐based materials [54].

### 2.2 Carbonization‐Hydrothermal/Heating Treatment

Hydrothermal synthesis is widely utilized in the fabrication of diverse carbon‐based materials due to its cost efficiency and the relatively moderate temperatures at which it operates [55]. This technique involves immersing the plants in a solution containing selected metal salts, such as those of Ni, Cu, or organic precursors. Following impregnation, the treated plants is subjected to high‐temperature pyrolysis, which induces the decomposition of the metal salts and promotes the formation of metal nanoparticles or oxide layers on or within the plant's surface.

Among various synthesis methods, the solvothermal approach offers several notable advantages [56]: (1) minimal post‐synthesis treatment and high product purity; (2) precise control over product morphology, structure, and composition; and (3) a mild and straightforward reaction process with an easily regulated reaction rate. Moreover, the solvothermal method facilitates the integration of electrocatalytically active materials onto plant‐based substrates. For instance, Gao et al. [48] utilized graphitized wood‐derived carbon (GWC) as a framework to develop dual‐active catalytic sites through the incorporation of Pt‐Ni recombination (Figure 3b). This innovative design enabled the modulation of interfacial atomic interactions through in situ phase transitions, thereby enhancing both the activity and durability of Pt nano catalysts for the hydrogen evolution reaction (HER). Initially, Ni(OH)_2_ nanosheets were synthesized on the GWC substrate. Subsequently, Pt nanoparticles were deposited in situ, resulting in the formation of Pt‐Ni(PO_3_)_2_‐GWC. This configuration significantly enhanced the efficiency of hydrogen production via water electrolysis [48].

Unfortunately, the solvothermal method generally requires an appropriate solvent and a sealed reaction vessel to sustain specific temperature and pressure conditions, governed by the vaporized solvent. These requirements contribute to elevated operational costs.

### 2.3 Carbonization‐Electrodeposition

Electrodeposition represents a versatile and efficient methodology for synthesizing plant‐supported electrocatalysts under mild conditions, offering a straightforward approach that enables controlled and effective material fabrication [57]. Through electrochemical reactions, metal ions are deposited onto the plant surface, enhancing both its conductivity and chemical stability [58]. Specifically, the process involves immersing a plant sample in an electrolyte solution containing targeted metal ions. Upon application of voltage, these ions undergo reduction at the wood surface, yielding a uniform metal coating. This process produces a conductive metal layer on the plant substrate, markedly improving the electrode material's overall performance.

Electrodeposition holds distinct advantages in the fabrication of self‐supported electrode catalysts. Notably, it is a simple, aqueous procedure that can be conducted under mild conditions within a relatively short timeframe. The morphology, loading, and composition of the electrocatalyst can be precisely controlled by adjusting key reaction parameters, such as deposition time, potential/current, and electrolyte composition. Wang et al. [49] used nickel‐cobalt layered double hydroxide (NiCo‐LDH) nanosheets to electrodeposit onto nitrogen‐doped carbonized wood (NCW) to fully utilize the tracheid space in the wood (Figure 3c). Wu and co‐workers [59] successfully developed ruthenium‐cobalt (RuCo) nanosheets supported on three‐dimensional porous carbon derived from pinewood (referred to as RuCo@TDC). This synthesis was achieved through a precisely optimized electrodeposition technique, resulting in a highly effective electrocatalyst for nitrate reduction reactions. Similarly, Li et al. [60] employed an in situ strategy to fabricate a free‐standing hierarchical carbon electrode by encapsulating nickel nanoparticles within nitrogen‐doped carbonized wood (Ni@NCW). The porous architecture of carbonized wood provides a unique scaffold that supports catalyst nucleation and active component loading, thereby facilitating efficient mass diffusion. The in situ incorporation of nitrogen‐doped carbon nanotubes can significantly enhance electron transfer within the electrode matrix and concurrently improve the stability of the active metal species [61]. By capitalizing on its hierarchical porous architecture and discrete active sites, this catalyst exhibits exceptional performance as a self‐supporting electrode for the HER in different pH environments, which affords the industrial‐level current density of 1000 mA cm^−2^ at a low overpotential of 401 mV, characterized by highly favorable kinetic properties.

### 2.4 Carbonization‐Chemical Vapor Deposition

Chemical vapor deposition (CVD) is an established and highly effective technique for depositing conductive thin films on wood surfaces. Within a controlled high‐temperature environment, the gaseous precursor undergoes decomposition, resulting in the formation of a uniform carbon film on the wood's surface. This deposited film not only exhibits remarkable electrical conductivity but also significantly enhances the mechanical properties of plant‐based electrodes [62]. The versatility of the CVD process allows for the treatment of substrates with varying shapes and sizes, while the catalyst formation process remains rapid and efficient. Notably, the plant substrate itself functions as a structure‐directing agent during catalyst preparation, facilitating uniform distribution of the catalytically active material across the substrate [63]. This approach achieves high utilization rates at a comparatively low cost, a distinct advantage when employing precious metal‐supported catalysts. Consequently, CVD is widely recognized as a cost‐effective, straightforward, and efficient approach for synthesizing plant‐supported, metal‐based electrocatalysts [64].

In a recent study, Yan et al. [50] proposed a simple, low‐energy and efficient method to anchor N/O co‐doped carbon nanotubes on the surfaces of CW substrates by in situ chemical vapor deposition (Figure 3d). Zhou et al. introduced an advanced methodology that combines the concurrent formation of active sites with the development of porous carbon networks, thereby enabling the fabrication of wood‐integrated air electrodes [65]. This approach entails the in situ growth of Co‐doped ZIF‐8 within the hierarchical pores of the wood, followed by simultaneous pyrolysis. The resulting air electrode consists of Co‐NRR active sites embedded within a hierarchical porous carbon network. This wood‐derived electrode retains the wood's inherent hierarchical porosity and incorporates Co‐N active sites, culminating in a Co‐N@ACS structure that demonstrates enhanced oxygen reduction reaction (ORR) activity in alkaline electrolytes.

To improve the stability of the anode in seawater electrolysis and mitigate chloride‐induced corrosion, Chen et al. developed a W‐doped NiFe sulfide (W‐NiFeS/WC) electrode supported on wood carbon for full seawater electrolysis using an impregnation‐sulfidation method [66]. During the impregnation phase, metal salts infiltrate the wood channels, facilitating their anchoring and adsorption onto the cell walls. Following this, high‐temperature pyrolysis converts the wood's intrinsic components—lignin, hemicellulose, and cellulose‐ into carbonized wood, while W‐NiFeS nanoparticles are uniformly and densely deposited on both the external and internal surfaces of the wood channels. This process yields a self‐supporting electrode characterized by high mechanical strength, significant porosity, and enhanced electrical conductivity.

### 2.5 Carbonization—Impregnation/Chemical Plating

The impregnation technique provides a straightforward and efficient approach for the synthesis of supported metal‐based electrocatalysts. Gan et al. [20] introduced a scalable and economical CW electrode for comprehensive water splitting by in situ decoration of Co/Ni binary nanoparticles within the CW channel (Co/Ni‐CW). Natural plants, rich in hydroxyl groups, serve as a versatile substrate, enabling the anchoring of metal cations within the cell wall through coordination bonds. High‐temperature calcination significantly enhances the formation of CW and Co/Ni binary nanoparticles. Hui and colleagues designed a 3D framework from natural wood and loaded it with an amorphous NiP alloy via a simple electroless plating technique to enhance the catalyst's HER performance in an alkaline solution (Figure 3e) [54].

Additionally, the 3D plants' aerogel has recently garnered significant interest, combining plants' renewable properties with aerogels' high porosity and low density [67]. This approach offers thermal insulation, adsorption, catalytic, and mechanical benefits. A novel layered bifunctional nanoelectrode design by Chen et al. [68] features a S, P‐(Ni, Mo, Fe)OOH electrocatalyst layer uniformly deposited on a NiMoP substrate over a channel‐rich plants aerogel, providing superior catalytic performance and stability when evaluated in alkaline seawater electrolytes.

### 2.6 Eco‐Friendly Synthesis Methods

It is worth noting that, several innovative and eco‐friendly synthesis methods are being developed to produce wood‐based electrodes while minimizing the environmental drawbacks of traditional high‐energy and chemical‐intensive processing [69]. One prominent green approach is the use of biological treatments, where white‐rot fungi are employed to selectively degrade lignin in the wood structure before carbonization [70]. This bioactivation method is highlighted as a safe, energy‐saving, and environmentally friendly alternative to harsh chemical or physical activation, as it operates under mild conditions with less energy consumption and avoids reactor corrosion. Another key innovation is the development of carbonization‐free, low‐temperature techniques, such as the steam‐driven self‐assembly of MXene onto delignified wood [71]. This strategy significantly simplifies traditional high‐temperature annealing processes, and life cycle assessments have confirmed it has a minimal impact on the environment, human health, and resource consumption. Furthermore, researchers are focusing on mild chemical modifications, such as using a simple and green sulfite process to functionalize and preserve the wood's native lignin, thereby utilizing it as a redox‐active component rather than treating it as a waste product from aggressive delignification [72]. These methods collectively demonstrate a shift towards more sustainable manufacturing by leveraging biological processes, reducing energy inputs, and maximizing the use of the wood's inherent components and structure. Briefly speaking, the one‐step carbonization method is a straightforward and cost‐effective approach suitable for large‐scale production of highly conductive electrode materials, though it is limited to pure carbon materials and challenges arise in incorporating multifunctional components. Hydrothermal or thermal treatments can introduce functional elements like oxides, phosphides, and sulfides into carbonized plants, enhancing electrocatalytic activity and tailoring specific electrochemical properties, albeit with increased costs. The carbonization‐electrodeposition method enables precise and uniform deposition of metals or oxides, allowing controlled material distribution for electrocatalytic and battery electrode applications, but it requires advanced equipment and process control. Similarly, carbonization‐CVD introduces functional films, such as nanomaterials, to significantly enhance catalyst surface performance, though its complexity and cost restrict its use to high‐value applications. Alternatively, carbonization‐impregnation or electroless plating offers a simpler, industrially scalable process for incorporating multiple functional components, provided the precursor solution and post‐processing are carefully optimized. Ultimately, wood‐based electrodes are an ideal green material, but their true environmental value depends on the preparation process. Selecting the appropriate method requires balancing application‐specific performance requirements against cost and technical constraints.

## 3 Features of Plant‐Based Monolithic Electrodes

In the typical preparation process of catalytic electrodes, powder catalysts are usually loaded onto electrode substrate materials with binders by spraying or dripping or grown in situ by hydrothermal methods and electrodeposition. However, low electrocatalyst utilization, poor gas/liquid transfer, and weak stability caused by physical contact prevent them from being used in industrial applications for a long time [73, 74]. To satisfy the demands for high site loading, superior conductivity, minimal ion diffusion resistance, and robust mechanical strength, the development and optimization of integrated electrode designs have garnered increasing attention [75]. Plants are favorable choice for carbon sequestration and carbon neutrality efforts due to their natural, renewable and abundant properties, as well as their environmentally friendly characteristics as a biomass material [76]. In particular, plants benefit from many neatly arranged vertical channels in nature, which are fast pathways for water, ions, and nutrient transport [64].

### 3.1 High Porosity

Plants can be characterized as a naturally occurring nanocomposite, comprising a multitude of cells—such as cell walls and lumens—largely aligned along the growth axis, with cellulose fibrils in a highly crystalline state. These fibrils are cohesively bound by interfibrillar compounds, including hemicellulose and lignin [19]. Hierarchical plant‐based structures can be readily derived from natural plants by delignification, such as sodium chlorite bleaching, which facilitates the selective removal of lignin. This process results in the near‐total separation of lignin from the plant cell wall, accompanied by minimal dissociation of cellulose and hemicellulose. Consequently, a porous structure with multidimensional anisotropy, extending across scales, from the macroscale (10^−2^ to 10^−4^ m) to microscale fibers (10^−4^ to 10^−7^ m), nanoscale elementary fibrils (10^−7^ to 10^−9^ m), and extending down to the molecule chains of cellulose (10^−9^ to 10^−10^ m), is preserved [77]. At cellular and tissue levels, the composition of plants varies significantly across species, exhibiting differences in both cell types and volume fractions, which influence the structure and proportions of its primary components.

The inherent porosity of plants renders it an advantageous material for use as an electrode. High porosity offers abundant active sites, which in turn expand the specific surface area of the electrode and enhance its electrochemical properties. In energy storage applications, particularly in supercapacitors and batteries, such porosity facilitates rapid ion and electron transport, thereby reducing charge transfer resistance and accelerating electrochemical reaction rates. Furthermore, this porosity allows greater penetration of electrolyte into the electrode interior, contributing to improved capacity and stability in electrochemical performance.

**FIGURE 4:**
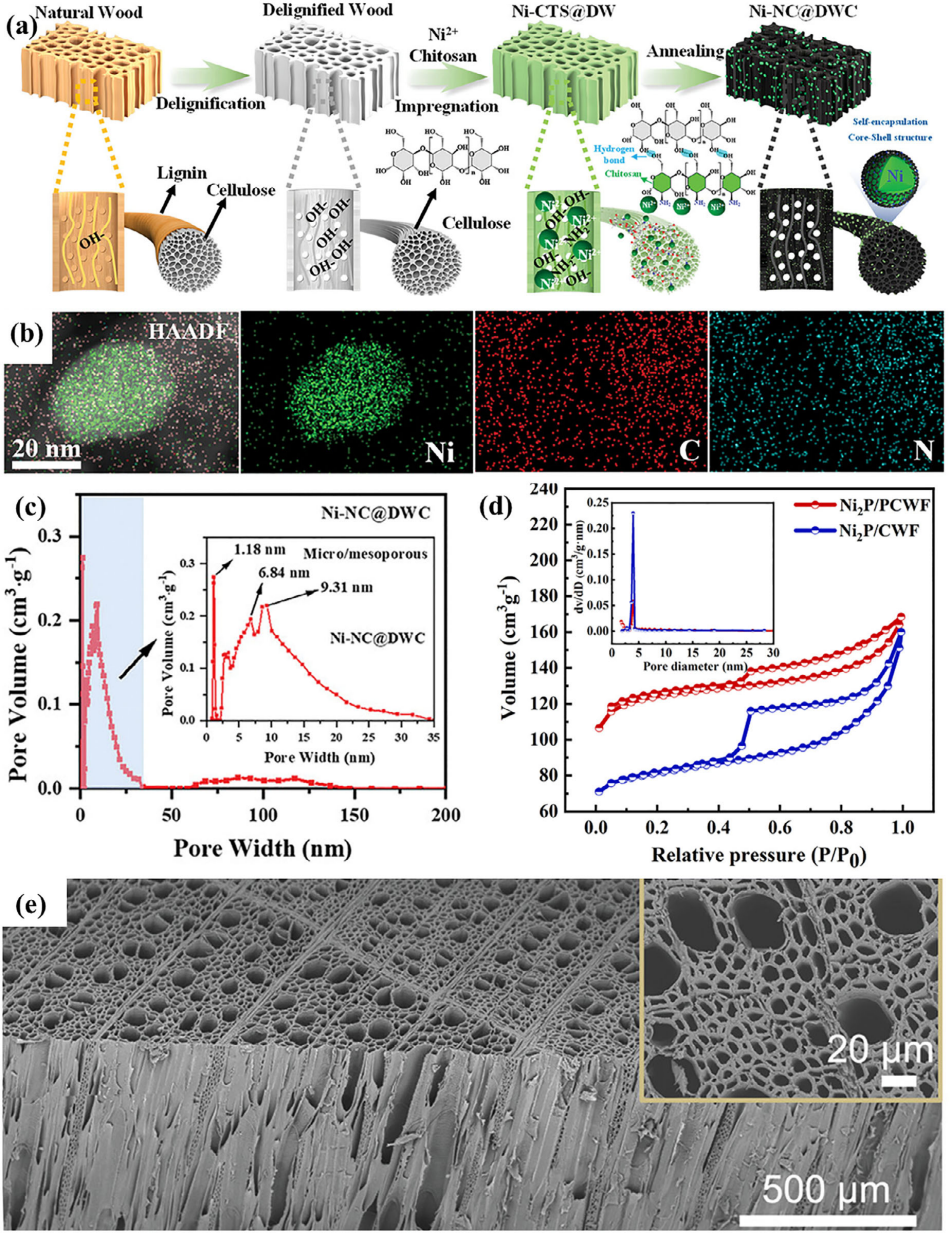
(a) Schematic illustration of the synthesis of Ni‐NC@DWC. (b) Corresponding elemental mappings of the Ni‐NC@DWC. (c) Corresponding pore size distribution. Reproduced with permission [78]. 2024 Wiley‐VCH. (d) BET N_2_ adsorption/desorption isotherms. Reproduced with permission [79]. Copyright 2022 Hydrogen Energy Publications LLC. (e) SEM image of natural wood after Co^2+^ and Ni^2+^ immersion. Reproduced with permission [54]. 2021 Wiley‐VCH.

Shi et al. developed an efficient, durable and self‐supporting electrocatalyst by encapsulating nickel nanoparticles in nitrogen‐doped carbon shells and growing them in situ on delignified woody carbon (Ni‐NC@DWC) (Figure 4a,b) [78]. The highly open, low‐tortuosity, 3D interconnected hierarchical porous structure of Ni‐NC@DWC is beneficial for the electron and electrolyte transport within the electrode, ensuring the rapid desorption of gaseous products. The pore size distribution of Ni‐NC@DWC reveals the dominance of micropores/mesopores, which enables the rapid diffusion of reactants to the active centers (Figure 4c). Similarly, Gan et al. [20] observed that Co‐Ni modification substantially increased the S_BET_ of Co/Ni‐CW to 216.12 m^2^ g^−1^ compared to unmodified CW (0.609 m^2^ g^−1^), underscoring the positive impact of Co/Ni incorporation on surface area enhancement. Kang et al. [79] used nitrogen adsorption‐desorption experiments to study the specific surface area and porous characteristics of Ni2P/PCWF (Figure 4d), verifying that the porosity of the catalyst is conducive to promoting mass transfer and enabling reactants to enter the active sites.

A scalable, low‐cost, and efficient CW electrode for overall water splitting was developed by in‐situ decoration with Co/Ni binary nanoparticles within CW channels (Co/Ni‐CW) [54]. The 3D porous structure consisting of multiple large channels (40–70 µm) and multiple small channels (10–25 µm) in natural basswood was well presented from the scanning electron microscope (SEM) image of the catalyst (Figure 4e). All wood channels were arranged straight from the top to the bottom of the wood, which enabled the Co and Ni ion solutions to penetrate into the entire wood and achieve the in‐situ formation of Co/Ni binary nanoparticles within the wood structure.

In contrast, Wang et al. report findings that diverge from previous conclusions, using BET analysis to assess the specific surface area of AuPd alloy nanoparticle‐modified CW. Their analysis reveals that the *S*_BET_ of Au_95_Pd_5_@CW (79.7 m^2^ g^−1^) is substantially lower than that of unmodified CW (112.1 m^2^ g^−1^), with total pore volume similarly halved due to the incorporation of alloy nanoparticles [80]. This reduction in surface area likely stems from the CW matrix's structural characteristics: its aligned channels and mesopores not only enable rapid electrolyte transport but also offer ample surface area for the dispersion of AuPd alloy nanoparticles. The observed decrease in surface area for Au_95_Pd_5_@CW is thus attributed to a reduction in external surface area, implying that metal ions predominantly deposit on the external surface.

### 3.2 Low Tortuosity

In the context of structural engineering, natural plants are widely esteemed for their low tortuosity and hierarchical porosity, which facilitate the formation of gradient pores and upright channels, essential for the efficient transport of water and nutrients. Owing to its biomimetic mass transfer capabilities, the porous structure of plants has inspired scientists to investigate monolithic electrodes that inherit these inherent low‐tortuosity channels, potentially enhancing the performance of electrochemical devices [31]. Beyond its advantageous porosity, plants' natural fibrous composition imparts a low bending modulus, contributing to its structural flexibility. This low tortuosity can effectively mitigate the risk of cracking or fracturing during use, thereby extending the device's operational lifespan and reliability.

A core characteristic of the aligned channel structure in plants is its minimized tortuosity, which ensures a shortened ion transport path, thus optimizing electrocatalytic efficiency [81]. For thicker plant‐based electrodes, the transport path through the pores becomes significantly extended, which may shift ion diffusion resistance beyond charge transfer resistance, potentially imposing a rate‐limiting constraint on the electrochemical reaction. Furthermore, due to the electrolyte concentration gradient and inherent limitations in ion transport, fully utilizing the active sites situated deeper within the plant structure proves challenging. The integrated conductive network and low‐tortuosity channels within plant‐based monolithic electrodes not only improve the embedding of active materials but also promote more efficient ion and electron transport. This improved conductivity and transport efficiency contribute to increased power and energy density in such electrochemical systems.

**FIGURE 5:**
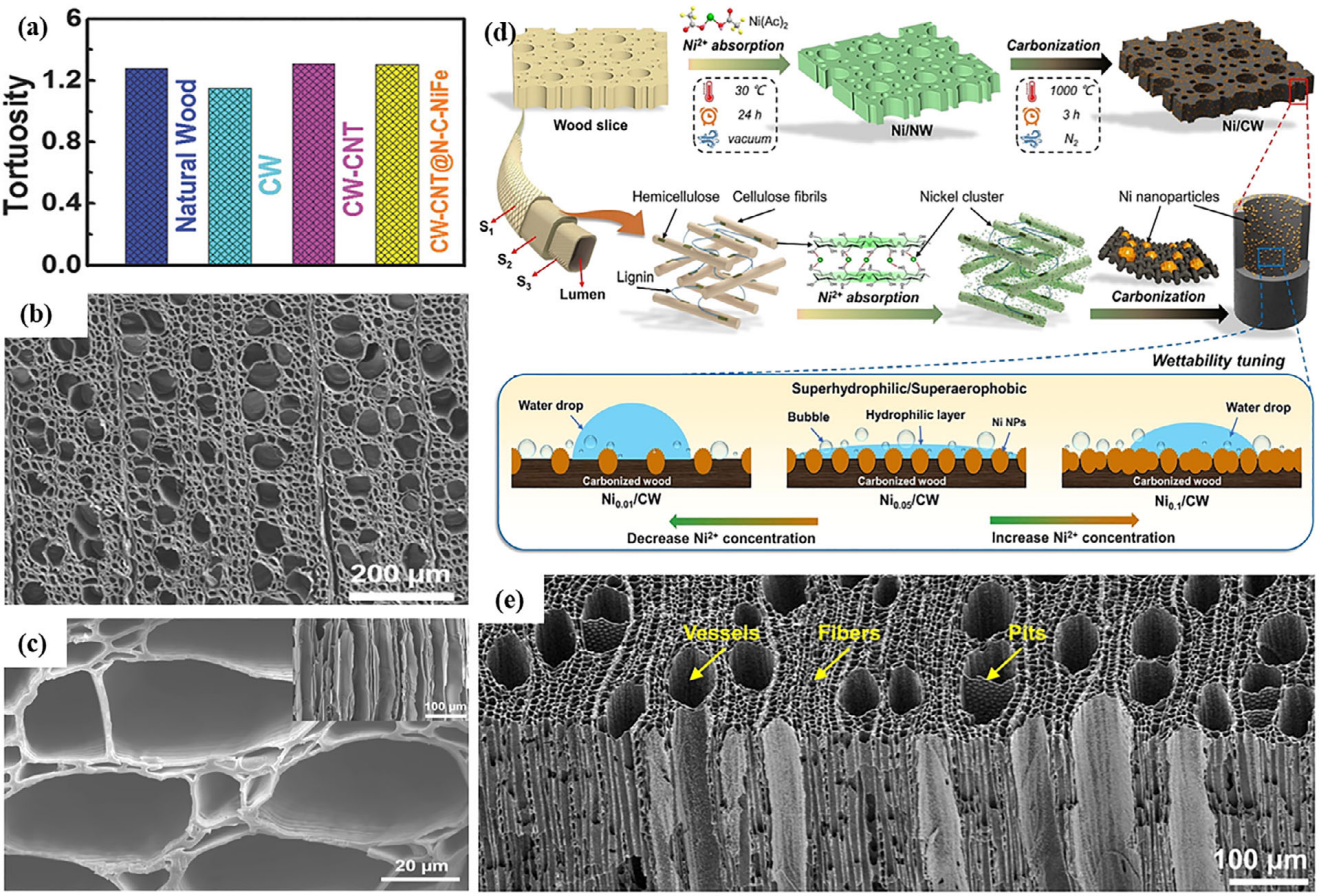
(a) Tortuosity values comparison for different wood‐based materials. (b) SEM image of the top surface of the natural wood, and (c) the carbonized wood. Reproduced with permission [82]. Copyright 2018 WILEY‐VCH. (d) Schematic representation of the fabrication process for the in situ integration of nickel nanoparticles into the carbonized wood matrix. (e) Overview SEM image. Reproduced with permission [83]. Copyright 2022 Chinese Academy of Sciences.

Li and colleagues compared the tortuosity of the heat‐shocked CW‐CNT@N‐C‐NiFe electrodes with other samples (Figure 5a) [82]. Their analysis indicates that the bare CW exhibited lower tortuosity values than both the CW‐CNT and CW‐CNT@N‐C‐NiFe electrodes, a difference attributable to the presence of CNTs and electrocatalysts within the microchannels. Notably, even the CW‐CNT@N‐C‐NiFe electrode displayed significantly lower tortuosity than conventional slurry‐coated electrodes (which typically range from 3 to 30). This structural characteristic promotes efficient electrolyte infiltration and facilitates the liberation of hydrogen gas bubbles. The morphology of the CW‐CNT@N‐C‐NiFe composite further corroborates this observation, as illustrated in Figure 5b,c. The wood retains its natural porous, low‐tortuosity microstructure along the growth axis.

Liao's group developed a straightforward and sustainable approach for producing low‐cost, efficient urea oxidation reaction (UOR) electrocatalysts. These catalysts comprised CW electrodes derived from poplar wood, with nanoparticles uniformly distributed within the channels, as shown in (Figure 5d) [83]. The CW framework, characterized by high graphitization, along with embedded Ni nanoparticle cores, augments electrode conductivity and improves the performance of the UOR. Wood's low‐tortuosity, hierarchically porous structure facilitates unrestricted mass transport pathways, exposing abundant active sites, which significantly improves UOR efficiency. SEM image reveals that the carbon morphology largely preserves the wood's original hierarchical architecture, displaying a 3D open‐pore structure with multiple large (50–100 µm) and small (5–20 µm) channels (Figure 5e). These microchannels feature low tortuosity and extend uniformly from the top to the bottom. This configuration facilitates enhanced electrolyte permeation, efficient gas product release, and a reduction in ion transport resistance.

Basswood, a representative hardwood with a complex, aligned channel structure, exhibits two types of pores within its cross‐section: smaller pores derived from cellulose fibers (<10 µm) and larger conduits (∼50 µm). Viewed along the growth direction, the aligned tubes extend through the entire trunk with minimal curvature. These vessel tubes (constituting ∼15% of basswood trunk volume) and fiber channels (∼30%) create ideal pathways for sustained water transport and filtration in the living tree. Following carbonization, this natural 3D scaffold structure supports effective electrode performance by enabling electrolyte ions to access nearly the entire surface of active sites, irrespective of electrode thickness [84].

### 3.3 High Electrical Conductivity

Conductivity, in general, refers to the rate at which electrons are transported through a material, which plays a critical role in determining electrode materials' performance. Wood‐derived electrodes offer several advantages, including natural renewability, favorable mechanical properties, and low cost. Furthermore, through carbonization and functionalization treatments, their conductivity and electrochemical properties can be significantly enhanced, positioning them as promising candidates for electrode materials [37, 85].

Studies have demonstrated that the influence of carbonization temperature on conductivity is significant. It is generally accepted that as the carbonization temperature increases, the carbon content of the wood electrode rises, the degree of graphitization enhances, and consequently, conductivity improves [86]. For instance, Gabhi et al. [87] found that as the carbon content of wood increased from 86.8% to 93.7%, its conductivity rose from 2.5 × 10^−4^ S m^−1^ to 399.7 S m^−1^, an increase of more than six orders of magnitude. In Wang et al.’s study, optimization of the carbonization temperature and wood thickness yielded carbonized wood with a conductivity of 2.2 S m^−1^. Their findings indicated that the optimal thickness for carbonized wood, considering both electron and ion transport capabilities, was 1.532 mm, resulting in optimal electrochemical performance in supercapacitors [88]. Additionally, functionalizing plant‐based materials is an effective strategy to enhance conductivity. Tran et al. introduced the conductive polymer poly(3,4‐ethylenedioxythiophene): polystyrene sulfonate (PEDOT: PSS) into sulfonated wood, which not only imparted conductivity but also facilitated strong electrostatic interactions with the sulfonated lignin component. This promoted tight coupling between the redox‐active lignin and the charge‐transporting PEDOT, resulting in a wood‐based electrode with a conductivity of up to 203 S m^−1^ [89]. Similarly, the lignin sulfonate in the wood structure acted as a dopant and stabilizer for the synthesized polypyrrole, achieving a high conductivity of 186 S m^−1^ for the composite material [40]. Carbon nanomaterials, such as carbon nanotubes and graphene quantum dots, have also been utilized to improve the conductivity of wood‐based electrodes. Wu et al. significantly enhanced the conductivity of wood‐based carbon materials by growing carbon nanotubes (CNTs) within them, leading to excellent rate performance in supercapacitors [90]. Graphene quantum dots‐modified wood‐based carbon electrodes have demonstrated improved electron transport and optimized charge storage pathways [91]. Metal and metal oxide modifications are also common approaches. Electrodes made from 3D layered wood (wood@Ni@Zn), constructed through electroplating Ni and subsequent electrochemical Zn deposition, exhibited high electrical conductivity (173.6 S m^−1^) and a long service life [92]. Similarly, the incorporation of metal oxides such as NiCo_2_S_4_ onto wood‐based carbon electrodes simultaneously improved conductivity and electrochemical activity [84]. These modification techniques collectively enhance the application potential of wood‐based electrodes.

Secondly, the distinctions between hardwoods and softwoods result in notable differences in their conductivity and electrochemical properties [93, 94]. Studies have indicated that hardwoods, such as African ironwood and olive wood, typically possess higher density and more intricate microstructures, which contribute to their enhanced conductivity. In contrast, softwoods, including pine and redwood, have lower density and simpler microstructures, resulting in comparatively lower conductivity. Bamboo, however, exhibits significantly higher conductivity than other types of wood, primarily due to its high cellulose content and larger graphite nanocrystal size. The conductivity of bamboo electrodes, after carbonization at 1500°C, reached 21,000 S m^−1^, which is substantially higher than that of other wood types [95].

It is important to note that the anisotropy of wood, such as differences between transverse and longitudinal cutting, significantly impacts its conductivity, with electrodes made from transversely cut wood exhibiting superior conductivity [96]. The conductivity of wood electrodes increases with the application of compression pressure, up to a certain point where internal cracks begin to form, causing a decrease in conductivity. Moreover, when the pressure is released, the conductivity returns to its pre‐compression level, a phenomenon referred to as the elastic behavior of the electric conductivity of biochar [87]. Although wood‐derived electrodes show great potential, optimizing their structure to further enhance conductivity and reduce internal resistance in practical applications remains a critical area of ongoing research and a significant challenge.

In summary, the natural high porosity of plant‐based materials provides a vast surface area with abundant active sites, promoting rapid ion and electron transport. Besides, their inherent structure of straight, aligned channels results in low tortuosity, which shortens ion transport paths and improves mass transfer efficiency. In addition, although initially poor conductors, their electrical conductivity can be significantly enhanced through treatments like high‐temperature carbonization and modification with conductive materials, making them a highly effective and sustainable platform for electrochemical applications (See Tables 1 and 2).

## 4 Plant‐Based Monolithic Electrodes for Small Molecule Conversion

### 4.1 Water Electrolysis

Water electrolysis, a process by which water is split into hydrogen and oxygen through the application of electrical energy, represents a promising avenue for sustainable hydrogen production [97, 98, 99]. The hydrogen generated by electrolysis serves as a clean energy vector with versatile applications, including fuel cells, and chemical synthesis, and as a potential replacement for fossil fuels [100, 101]. The development and optimization of highly active and stable electrocatalysts are essential for addressing the energy crisis and mitigating environmental pollution. Leveraging their rich hierarchical porous structures, high specific surface area, adjustable surface and interface properties, and uniformly dispersed active sites (such as metal nanoparticles, metal compounds, and heterogeneous catalytic structures), plant‐based nanomaterials are emerging as highly attractive electrocatalysts for the HER and oxygen evolution reaction (OER) [102].

**TABLE 1:** Summary of parameters for various plant‐based materials in water electrolysis.

Synthesis methods	Catalysts	Pore structure	Performance	Ref.
Carbonization‐hydrothermal treatment	CoP@PCM	*S*_BET_ = 229.4 m^2^ g^−1^	HER: *η*_10_ = 155 mV, 0.5 m H_2_SO_4_	[103]
	Co@N‐HPMC	*S*_BET_ = 249 m^2^ g^−1^, *S*_average_ = ∼4 nm, *V*_p_ = 0.249 cm^3^ g^−1^	HER: *η*_10_ = 128 mV, 1.0 m KOH OER: *η*_10_ = 297 mV, 1.0 m KOH	[104]
	Pt‐Ni(PO_3_)_2_ ‐GWC	—	HER: *η*_10_ = 10 mV	[48]
	Mo‐Ni_3_S_4_ /CW	*S*_BET_ = 59 m^2^ g^−1^,	HER: *η*_10_ = 17 mV, 1.0 m KOH OER: *η*_10_ = 240 mV, 1.0 m KOH	[105]
	NSP‐CF@NCW	Mean diameter 8.6 µm	UOR: 1.49 V versus RHE @ 50 mA cm^−2^	[106]
	Ni_3_Fe‐CW	—	HER: *η*_10_ = 76 mV, 1.0 m KOH OER: *η*_10_ = 237 mV, 1.0 m KOH	[107]
	CoNiNP‐N@CoNi/CW	*S*_BET_ = 375.65 m^2^ g^−1^, *S*_average_ = 8.35 nm, *V*_p_ = 0.22 cm^3^ g^−1^	HER: *η*_10_ = 143 mV, 1.0 m KOH	[108]
	NP MoS_2_/CW	—	HER: *η*_10_ = 109.5 mV, 0.5 m H_2_SO_4_	[109]
	NiS/Ni_3_S_4_/GCW	*S*_BET_ = 62.5 m^2^ g^−1^	HER: *η*_10_ = 91 mV, 1.0 m KOH	[110]
	Fe/Fe_3_C/CNT@MoS_2_@GW	*S*_BET_ = 422.689 m^2^ g^−1^	HER: *η*_10_ = 63 mV, 0.5 m H_2_SO_4_	[111]
	NiFe‐LDHs@NiFe/CW	*S*_BET_ = 193.82 m^2^ g^−1^	OER: *η*_50_ = 212 mV, 1.0 m KOH	[112]
	Ni_2_P/PCWF	*S*_BET_ = 473.6 m^2^ g^−1^, *S*_average_ = 4 nm, *V*_p_ = 0.25 cm^3^ g^−1^	UOR: 1.34 V versus RHE @ 50 mA cm^−2^	[79]
Carbonization‐CVD	W‐NiFeS/WC	Macropores 10−60 µm in diameter	Seawater: 1.673 V @ 100 mA cm^−2^, *E*_corrosion_ = 0.258 V versus RHE, *J*_corrosion_ = 6.341 µA cm^−2^, 1.0 versus KOH	[66]
	MoS_2_‐MoO_2_/CW	—	HER: *η*_10_ = 148 mV, 0.5 m H_2_SO_4_	[113]
Carbonization‐electrodeposition	Ni@NCW	—	HER: *η*_1000_ = 401 mV, 0.5 m H_2_SO_4_	[60]
	GO/Ni‐Co/CW	—	HER: *η*_10_ = 52 mV, 0.5 m H_2_SO_4_	[57]
	a‐NiFe@Ni‐CW	*S*_BET_ = 157 m^2^ g^−1^	OER: *η*_100_ = 268 mV, 1.0 m KOH	[114]
	Pd‐NiS/CW	—	HER: *η*_10_ = 80 mV, 1.0 m KOH	[115]
Carbonization‐impregnation	Ni_0.05_/CW	Large and small pores are 50–100 µm and 5–20 µm	UOR: 1.36 V versus RHE @ 10 mA cm^−2^	[83]
	Co/Ni‐CW	Large and small pores are 40–70 µm and 10–25 µm, *S*_BET_ = 216.12 m^2^ g^−1^, *S*_average_ = 3.851 nm, *V*_p_ = 0.220 cm^3^ g^−1^	HER: *η*_10_ = 157 mV, 1.0 m KOH OER: *η*_10_ = 330 mV, 1.0 m KOH	[20]
Others	S,P‐(Ni,Mo,Fe)OOH/NiMoP/wood aerogel	Large and small pores are 200–300 µm and 15–80 µm	HER: *η*_500_ = 258 mV, 1.0 m KOH OER: *η*_500_ = 297 mV, 1.0 m KOH	[68]
	HEAs@ACW	—	HER: *η*_10_ = 7 mV, 1.0 m KOH	[116]
	Mo_2_N‐Mo_2_C/N‐CW	—	HER: *η*_10_ = 79 mV, 0.5 m H_2_SO_4_	[117]
	NCF_0.5_P@CNT/CW	*S*_BET_ = 352.6 m^2^ g^−1^	HER: *η*_50_ = 205 mV, 1.0 m KOH OER: *η*_50_ = 212 mV, 1.0 m KOH	[118]
	3D Ni‐W‐B/wood	—	HER: *η*_50_ = 46 mV, 1.0 m KOH	[119]
	CoP@PNC/PCWF	—	UOR: 1.5 V versus RHE @ 50 mA cm^−2^	[120]
	CW‐CNT@N‐C‐NiFe	—	HER: *η*_10_ = 179 mV, 0.5 m H_2_SO_4_	[82]

Abbreviations: *E*_corrosion_, corrosion potential; GO, graphene oxide; GWC, graphitized wood‐derived carbon; HPMC, hierarchically porous monolithic carbon; PCM, porous carbon membrane.

**FIGURE 6:**
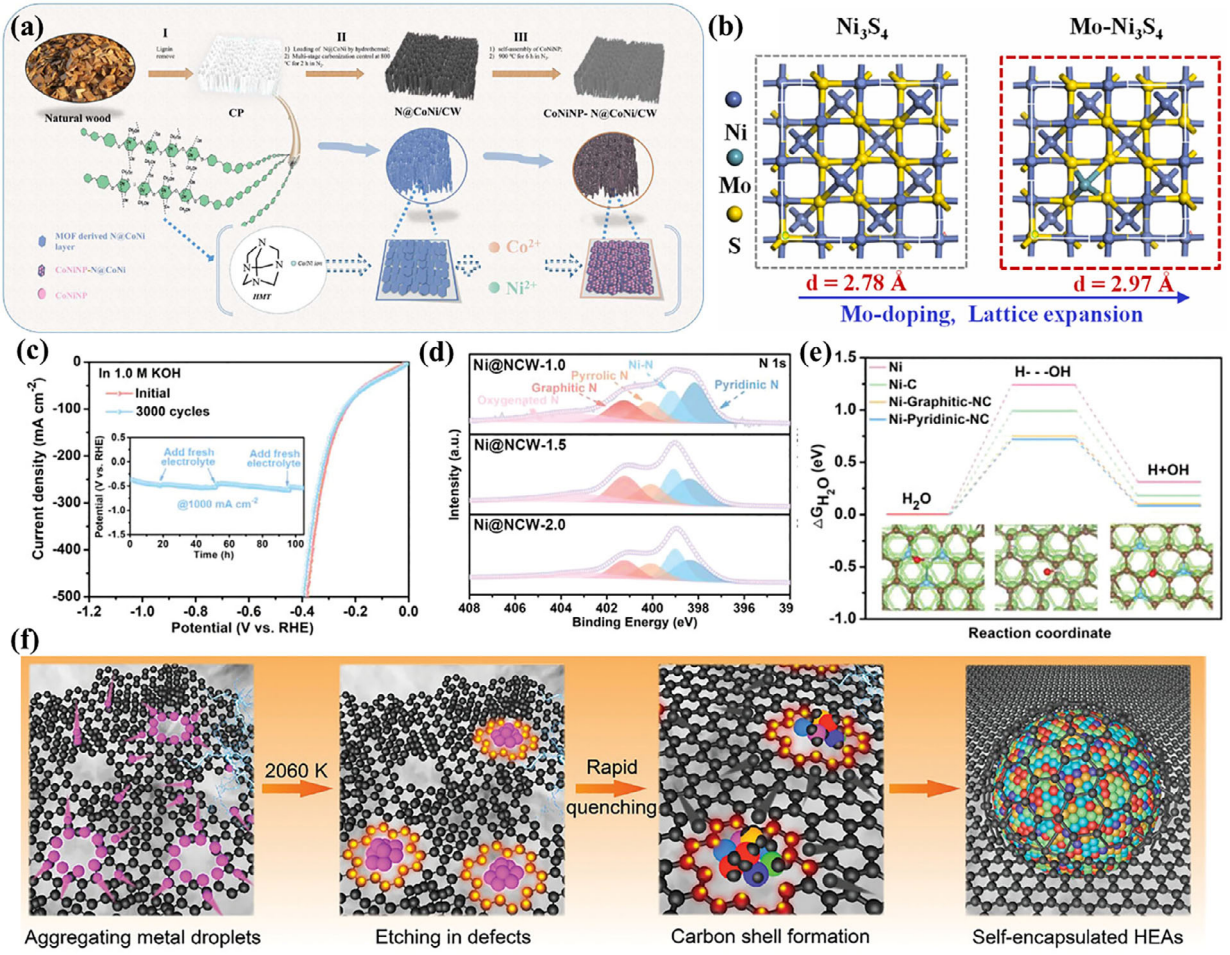
(a) Schematic illustration of the synthesis of CoNiNP‐N@CoNi/CW. Reproduced with permission [108]. Copyright 2022 Hydrogen Energy Publications LLC. (b) Structural models of Ni_3_S_4_ and Mo‐Ni_3_S_4_. Reproduced with permission [105]. Copyright 2023 Elsevier B.V. (c) Cycling performance of the Ni@NCW catalyst evaluated in a 1 m KOH solution. (d) High‐resolution XPS spectra of the N 1s orbital for the Ni@NCW catalysts. (e) Calculated reaction free energy for H_2_O dissociation on various optimized model surfaces. Reproduced with permission. [60] Copyright 2023 Wiley‐VCH GmbH. (f) Mechanism of the self‐encapsulation of HEAs driven by defect‐driven surface engineering. Reproduced with permission. [116] Copyright 2024 Wiley‐VCH GmbH.

The development of self‐supporting, nanoparticle‐embedded, plant‐derived porous carbon materials has become an effective and durable approach for electrocatalysis in the HER under both acidic and alkaline environments [121, 122]. The hydrothermal method facilitates the controlled deposition and dispersion of nanoparticles onto the plant's surface, enabling the construction of multi‐scale structures. This approach optimizes the pore architecture and surface morphology of plant‐derived carbon materials, thereby enhancing their catalytic properties. For example, Li et al. [104] utilized a two‐step carbonization process to fabricate monolithic porous carbon‐based electrodes modified with Co/CoO*_x_* NW sheets. These electrodes exhibited excellent catalytic activity and stability for HER and OER, as well as their coupled overall water‐splitting reactions in an alkaline environment (Figure 6a). Similarly, Shi and colleagues incorporated molybdenum into the Ni_3_S_4_ lattice grown on carbonized wood (denoted as Mo‐ Ni_3_S_4_/CW) to optimize hydrogen and oxygen species adsorption energies and modulate local charge densities at active sites, significantly enhancing both HER and OER performance (Figure 6b) [105]. Gao et al. advanced the field by employing GWC as a platform to construct dual‐active catalytic sites through Pt‐Ni recombination, leveraging in situ phase transitions to modulate heterointerface atomic interactions and thereby enhance the activity and durability of Pt‐based nanocatalysts for HER [48]. Furthermore, research has demonstrated that the establishment of a heterointerface between two electroactive phases can alter the electronic environment at the interface [123]. This modification occurs through the redistribution of electrons, which, in turn, increases the density of catalytically active sites. This, in turn, can significantly improve electrocatalytic efficiency [124]. Therefore, Farithkhan et al. [106]. were the pioneers in demonstrating the integration of 3D Se‐ and P‐modified NiSeP (NSP) microflakes with CuFe (CF) composite cubes, which were directly embedded within the multi‐channel structures of nitrogen‐doped carbonized wood scaffolds (NSP‐CF@NCW). They investigated how microstructural engineering of electronic frameworks and catalytic sites influences multifaceted enzyme‐free urea sensors and urea‐assisted water electrolysis. Notably, the NSP‐CF@NCW electrode achieved a current density of 50 mA cm^−2^ at a potential of 1.49 V versus RHE, representing a 210 mV reduction compared to the overpotential (*η*) typically required for conventional alkaline water‐splitting reactions.

Carbonization‐electrodeposition and carbonization‐impregnation are widely employed methods for fabricating plant‐based electrodes. Transition metal compounds, including Fe, Co, and Ni, as well as their nitrides, phosphides, and hydroxides, have been effectively integrated with conductive matrices to serve as bifunctional catalysts in water splitting and urea electrolysis applications [125, 126]. Traditional electrode substrates primarily consist of metal foams, carbon cloths, and semiconductors [124]. However, extending the use of substrates to sustainable natural plants presents an ideal avenue for producing high‐performance electrodes [20]. For instance, Li and colleagues developed a freestanding hierarchical carbon electrode by encapsulating nickel nanoparticles within nitrogen‐doped carbonized wood (Ni@NCW) through a straightforward in situ strategy. This electrode demonstrated exceptional HER electrocatalytic performance, achieving industrial‐grade current densities of up to 1000 mA cm^−2^ in 1 m KOH (Figure 6c) [60]. Serves a dual function in catalysis: it modulates the electronic properties of the catalyst while facilitating charge transfer. This synergistic effect enhances the material's conductivity, thereby markedly improving its catalytic performance in the HER (Figure 6d). The optimized water dissociation energy barrier and Δ*GH* ^*^ suggest that the synergistic interaction between Ni nanoparticles and the N‐doped carbon layer significantly enhances HER performance (Figure 6e). Similarly, integrated electrodes with tunable wettability, derived from hierarchical porous wood scaffolds, have shown promise for UOR [83].

High‐entropy alloys (HEAs) with entropy stabilization properties offer significant advantages for enhancing chemical stability and lowering thermodynamic energy barriers in HER due to their highly disordered structures and randomly distributed unsaturated sites [127]. However, like other nanocatalysts, HEAs face critical challenges in surface architecture during electrocatalysis, including nanoparticle detachment, inefficient electron transfer, and limited accessibility to active sites [128, 129]. Wang et al. demonstrated a defect‐driven surface engineering strategy, developing a highly active and durable electrocatalyst comprising five‐element HEAs (PtNiCoFeCu) encapsulated within activated carbonized wood (ACW) (Figure 6f). Unlike conventional pyrolysis methods that yield exposed structures, the self‐encapsulation of HEAs, stabilized by intrinsic defects and a protective carbon coating, resists tensile forces generated by cyclic bubble adsorption and desorption, thereby significantly improving structural stability during HER [116]. In addition, conventional preparation methods for chain metal catalysts often involve prolonged high‐temperature calcination and cooling phases, where slow heating and cooling rates frequently lead to nanoparticle agglomeration and degradation [130, 131]. Thermal shock treatment has emerged as a novel material synthesis technique, gaining attention for its distinct synthesis mechanisms, tunable physicochemical properties, and In a groundbreaking study, Li and colleagues introduced an innovative thermal shock methodology for the expedited synthesis of self‐assembled catenary‐structured nanocatalysts (N‐C‐NiFe) within an open‐channel, low‐torsion carbon nanotube network (CW‐CNT) [82]. The resultant freestanding CW‐CNT@N‐C‐NiFe composite electrode exhibited exceptional electrocatalytic characteristics and demonstrated prolonged durability in hydrogen evolution applications. Specifically, the electrode manifested a Tafel slope of 52.8 mV dec^−1^ and required merely 179 mV overpotential to achieve a current density of 10 mA cm^−2^. The thermal shock technique, characterized by instantaneous heating and rapid cooling cycles, effectively suppressed nanoparticle coalescence and migration, resulting in the generation of monodispersed, nanoscale catalytic particles. Furthermore, the unique architectural configuration of the CW‐CNT matrix, featuring vertically aligned microporous channels, enhanced electrolyte permeation and optimized hydrogen gas liberation kinetics.

Plant‐based monolithic electrodes exhibit exceptional performance in water splitting, particularly in the OER, which frequently serves as the rate‐determining step in the electrolysis process. The carbonized plants' inherently high surface area and porosity significantly enhance hydrogen evolution at the cathode during the HER. These properties provide abundant catalytic sites and facilitate efficient ion transport, thereby reducing the energy requirements for electrolysis and augmenting the overall efficiency of hydrogen production. By harnessing the unique porous architecture and mechanical resilience of plants, coupled with state‐of‐the‐art carbonization and functionalization methodologies, these electrodes achieve remarkable performance in both the OER and HER.

### 4.2 Oxygen Reduction

The oxygen reduction reaction is a pivotal process in energy conversion systems, including fuel cells and metal‐air batteries [132]. However, its inherently sluggish reaction kinetics presents a significant bottleneck for advancing next‐generation energy conversion technologies [131, 133]. To overcome this limitation, an integrated design approach that concurrently develops active catalytic sites and establishes porous carbon networks has been proposed. This strategy enhances charge transfer, mass transport, and electron conduction, thereby addressing the critical kinetic challenges of ORR.

**TABLE 2:** Summary of parameters for various plant‐based materials in oxygen reduction.

Synthesis methods	Catalysts	Pore structure	Performance (vs. RHE)	Ref.
Carbonization‐impregnation	Co‐N@ACS	—	*E*_1/2_ = 0.86 V	[65]
Carbonization‐hydrothermal treatment	TARC‐N	*S*_BET_ = 1438 m^2^ g ^−1^	*E*_initial_ = 0.98 V, *E*_1/2_ = 0.86 V	[134]
	Fe‐N‐C_wood_	*S*_BET_ = 840.9 m^2^ g^−1^	*E*_initial_ = 0.98 V, *E*_1/2_ = 0.9 V	[135]
	FeP‐NWCC	*S*_BET_ = 565 m^2^ g^−1^	*E*_1/2_ = 0.86 V	[136]
	NiCo_1.8_Fe_0.2_O_4_@N‐carbon	—	*E*_1/2_ = 0.86 V	[137]
	Fe_2_O_3_‐CW_1000_	*S*_BET_ = 505.95 m^2^ g^−1^	*E*_initial_ = 0.98 V, *E*_1/2_ = 0.78 V	[138]
Freeze‐carbonization‐impregnation	FeP@N,P‐WCA	Pore diameters of 30–50 µm, mesopore sizes: 3–5 nm, *S*_BET_ = 584.2 m^2^ g^−1^	*E*_initial_ = 0.95 V, *E*_1/2_ = 0.84 V, *I*_limit_ = 5.20 mA cm^−2^	[67]
	PCLFW@Li‐Na	—	*E*_initial_ = −0.12 V, *E*_1/2_ = −0.22 V	[139]
Others	Co@NCW	*S*_BET_ = 722 m^2^ g^−1^, *V*_p_ = 0.37 cm^3^ g^−1^	*E*_1/2_ = 0.89 V	[140]
	Bamboo charcoal	—	*E*_initial_ = −0.88 V versus Ag/AgCl	[141]
	NPBC	*S*_BET_ = 745.12 m^2^ g^−1^, *S*_average_ = 2.72 nm, *V*_p_ = 0.51 cm^3^ g^−1^	*E*_1/2_ = 0.864 V	[142]

Abbreviations: ACS, activated carbon substrate; *E*_1/2_, half‐wave potential; *E*_initial_, initial potential; NPBC, N‐doped pre‐treated bamboo‐based hierarchical porous carbon; PCLFW, porous carbonized lignin‐free wood; WCA, wood‐derived carbon aerogel.

**FIGURE 7:**
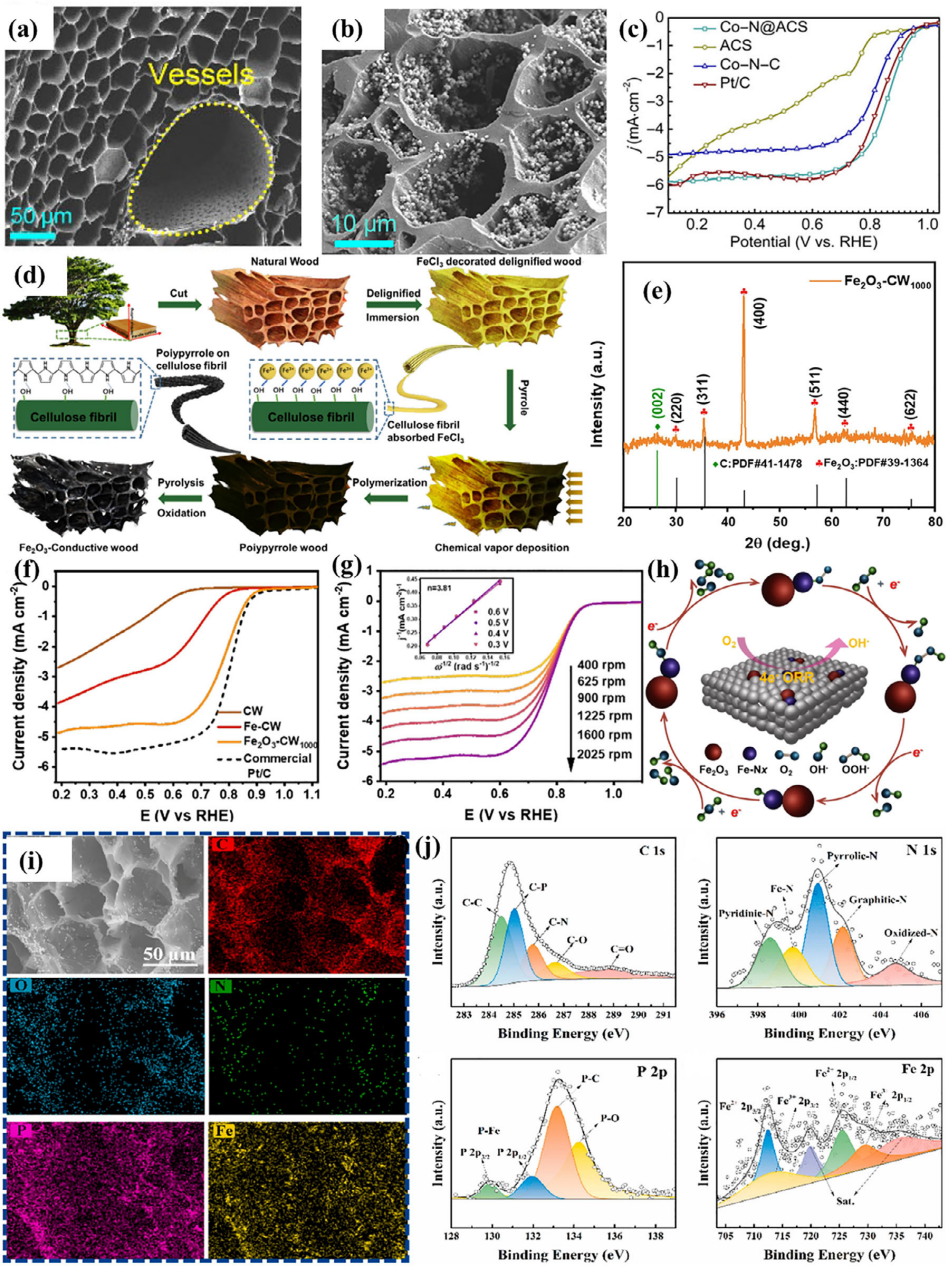
(a) SEM of the top‐view and cross‐sectional morphology of AWS and Co/ZIF‐8@AWS, and (b) Co‐N@ACS. (c) LSV curves of ACS, Co‐N‐C, Co‐N@ACS and 20% Pt/C at 1,600 rpm. Reproduced with permission [65]. Copyright 2021 Springer Nature. (d) Schematic illustration of the preparation procedures for Fe_2_O_3_‐CW_1000_. (e) XRD pattern of Fe_2_O_3_‐CW_1000_. (f) LSV curves of CW, Fe‐CW, Fe_2_O_3_‐CW_1000_, and commercial Pt/C. (g) LSV curves of Fe_2_O_3_‐CW_1000_ at different rotating speeds. (h) The catalytic mechanism of Fe_2_O_3_‐CW_1000_ sample for the ORR process in alkaline media. Reproduced with permission [138]. Copyright 2022 Elsevier B.V. (i) SEM image of FeP@N, P‐WCA and corresponding element mappings. (j) High‐resolution C 1s, N 1s, P 2p, Fe 2p XPS spectra of FeP@N, P‐WCA. Reproduced with permission [67]. Copyright 2024 Elsevier Ltd.

Platinum‐based catalysts, recognized as the most advanced cathode electrode catalytic materials, significantly enhance the sluggish kinetics of the ORR. However, their widespread adoption remains constrained due to the high cost, limited availability, and inadequate stability of precious metals. Addressing these challenges has prompted a general shift towards developing efficient and durable non‐precious metal alternatives to replace Pt‐based catalysts. Zhou et al. [65] introduced an integrated air electrode, termed Co‐N@ACS, which incorporates Co‐N sites and hierarchical porous carbon, synthesized by growing Co‐doped ZIF‐8 within an activated wood matrix (Paulownia) and simultaneously subjecting it to pyrolysis. The optimized electrode demonstrated exceptional ORR activity, with a half‐wave potential (E_1/2_) of 0.86 V versus RHE. SEM revealed that pretreatment induced the formation of numerous artificial pores on the vessel walls and channels, with ZIF particles distinctly anchored along these structures in the Co/ZIF‐8@AWS (activated wood substrates) framework (Figure 7a). Post‐pyrolysis, the hierarchical porous architecture of the wood substrate was well preserved, and the polyhedral, rough‐textured pyrolyzed ZIF was seamlessly incorporated into the Co‐N@ACS matrix (Figure 7b). The high E_1/2_ values and substantial limiting current densities further confirmed the superior ORR catalytic performance of Co‐N@ACS (Figure 7c).

In addition, heteroatom‐doped carbon materials have gained significant attention as promising metal‐free catalysts for the ORR, owing to their competitive electrochemical activity, superior conductivity, cost‐effectiveness, and remarkable durability [143, 144, 145, 146]. Miao and co‐workers [138] successfully prepared Fe_2_O_3_‐CW_1000_ using low‐cost nitrogen‐doped carbon from wood to support Fe^3+^ through a simple pyrolysis and oxidation method (Figure 7d). The XRD analysis confirmed the successful deposition of γ‐Fe_2_O_3_ on the CW substrate, with the characteristic peak at 26.38° demonstrating peak broadening, suggesting structural imperfections within the carbon matrix that potentially serve as catalytic centers for oxygen electrode reactions (Figure 7e). The Fe_2_O_3_‐CW_1000_ composite demonstrated superior electrocatalytic properties, particularly evidenced by its elevated onset potential (*E*_onset_) of 0.98 V, surpassing conventional commercial catalysts. This enhanced performance is attributed to the synergistic interaction between γ‐Fe_2_O_3_ and Fe‐N*_x_* species, which collectively promote ORR activity (Figure 7f). Furthermore, comprehensive investigations into the electron transfer mechanisms of Fe_2_O_3_‐CW_1000_, through both computational modeling and experimental validation, established that the ORR predominantly follows a four‐electron transfer pathway. These findings provide critical insights into the underlying mechanisms responsible for the improved ORR efficiency (Figure 7g,h).

An innovative approach for synthesizing highly active and durable oxygen electrocatalysts involves the deposition of transition metal compounds onto highly porous carbon aerogels. Compared to conventional catalyst supports, carbon aerogels offer tunability and structural control, enabling precise catalyst design and optimization to enhance activity and stability. Xu et al. [67] developed an innovative approach for cell wall nanoengineering, enabling the transformation of natural balsa wood into wood‐derived carbon aerogels (WCA). They further embedded FeP nanoparticles within hierarchically N, P‐doped WCAs, which were subsequently evaluated for their efficacy as ORR electrocatalysts. SEM and energy‐dispersive X‐ray spectroscopy (EDS) analyses provided critical insights into both the elemental composition and the spatial distribution within the FeP@N, P‐WCA. These factors are essential determinants influencing catalytic performance (Figure 7i). X‐ray photoelectron spectroscopy (XPS) was utilized to gain comprehensive insights into the surface chemical composition and electronic states of FeP@N, P‐WCA (Figure 7j). Five distinct nitrogen configurations were identified: pyridinic N (398.5 eV), pyrrolic N (400.9 eV), graphitic N (402.1 eV), Fe‐N (399.7 eV), and oxidized N (404.7 eV). Additionally, the presence of peaks at 133.2 eV (P‐C) and 134.2 eV (P‐O) further corroborated the successful integration of phosphorus into the carbon matrix. This modification notably improved the catalytic activity and stability of the material, extending its potential for a range of applications.

Plant‐based electrodes, particularly those functionalized with metal catalysts, represent an efficient and cost‐effective alternative to traditional platinum‐based cathodes. Their high surface area and porous architecture promote efficient oxygen diffusion, while the carbonized framework ensures excellent electrical conductivity for electron transfer. While plant‐based monolithic electrodes show promise for ORR applications, challenges such as scaling production and improving stability under harsh conditions remain.

### 4.3 CO_2_/Nitrate Reduction

At present, there are relatively few studies on plant‐based electrodes in CO_2_ and nitrate reduction, but some work has shown that good catalytic performance can be achieved through the functional treatment of plants. Some representative plant‐based materials for carbon dioxide/nitrate reduction are summarized in Table 3.

**TABLE 3:** Summary of parameters for various plant‐based materials in CO_2_/nitrate reduction.

Synthesis method	Electrode	Pore structure	Performance (vs. RHE)	Ref.
**CO_2_RR**				
One‐step carbonization	N‐CWM	Pore size = 5–50 µm, *S*_BET_ = 877.44 m^2^ g ^−1^, *V*_p_ = 0.49 cm^3^ g^−1^	FE_CO_ = 78% at −0.68 V	[147]
	Activated wood	*S*_BET_ = 182 m^2^ g^−1^	*J* = −53.8 mA cm^−2^, FE_HCOOH_ = 70.8% at −1.8 V (vs. SCE)	[148]
Carbonization‐impregnation	Ni SAs‐NCW	Pore size = 2.4 nm, *S*_BET_ = 1331.9 m^2^ g ^−1^, *V*_p_ = 0.78 cm^3^ g^−1^	*J* = −11.4 mA cm^−2^, FE_CO_ = 92.1% at −0.46 V	[149]
	Au_95_Pd_5_@CW	Channels = 5–80 µm; *S*_BET_ = 79.7 m^2^ g^−1^	FE_CO_ = 82% at −0.6 V	[80]
	Ag@CW‐ER	*S*_BET_ = 26.1 m^2^ g^−1^, *V*_p_ = 0.02 cm^3^ g^−1^	*J* = −22.6 mA cm^−2^, FE_CO_ = 88.6% at −0.97 V	[150]
Others	HDPCM	*S*_BET_ = 888.9 m^2^ g^−1^, *V*_p_ = 0.54 cm^3^ g^−1^	*J* = −3.88 mA cm^−2^, FE_CO_ = 81.1% at −0.66 V	[151]
**NRR**				
Carbonization‐impregnation	Cu_5_Co_5_/OMC	*S*_BET_ = 706.8 m^2^ g^−1^	*Y*_NH3_ = 21.05 mg h^−1^ cm^−2^, FE_NH3_ = 96.9% at −0.6 V	[152]
One‐step carbonization	Charcoal electrode	10 µm channel	*Y*_NH3_ = 0.570 mmol L^−1^ h^−1^ cm^−2^ at −3.6 V versus Hg/Hg_2_SO_4_	[46]
Carbonization‐electrodeposition	RuCo@TDC	—	*Y*_NH3_ = 2.02 ± 0.11 mmol h^−1^ cm^−2^ at −0.6 V, FE_NH3_ = 95.7 ± 0.8 % at −0.2 V	[59]
Carbonization‐hydrothermal treatment	Fe‐Co_3_O_4_/PC	—	*Y*_NH3_ = 0.55 mmol·h^−1^·cm^−2^, FE_NH3_ = 96.5% at −0.5 V	[153]

Abbreviations: ER, embedding reconstructed; FE_CO_, faradic efficiency of CO; HDPCM, highly defective porous carbon membrane; *J*, current density; OMC, ordered mesoporous carbon; PC, pinewood‐derived carbon; TDC, three‐dimensional porous carbon; *Y*_NH3_, yield of NH_3_.

**FIGURE 8:**
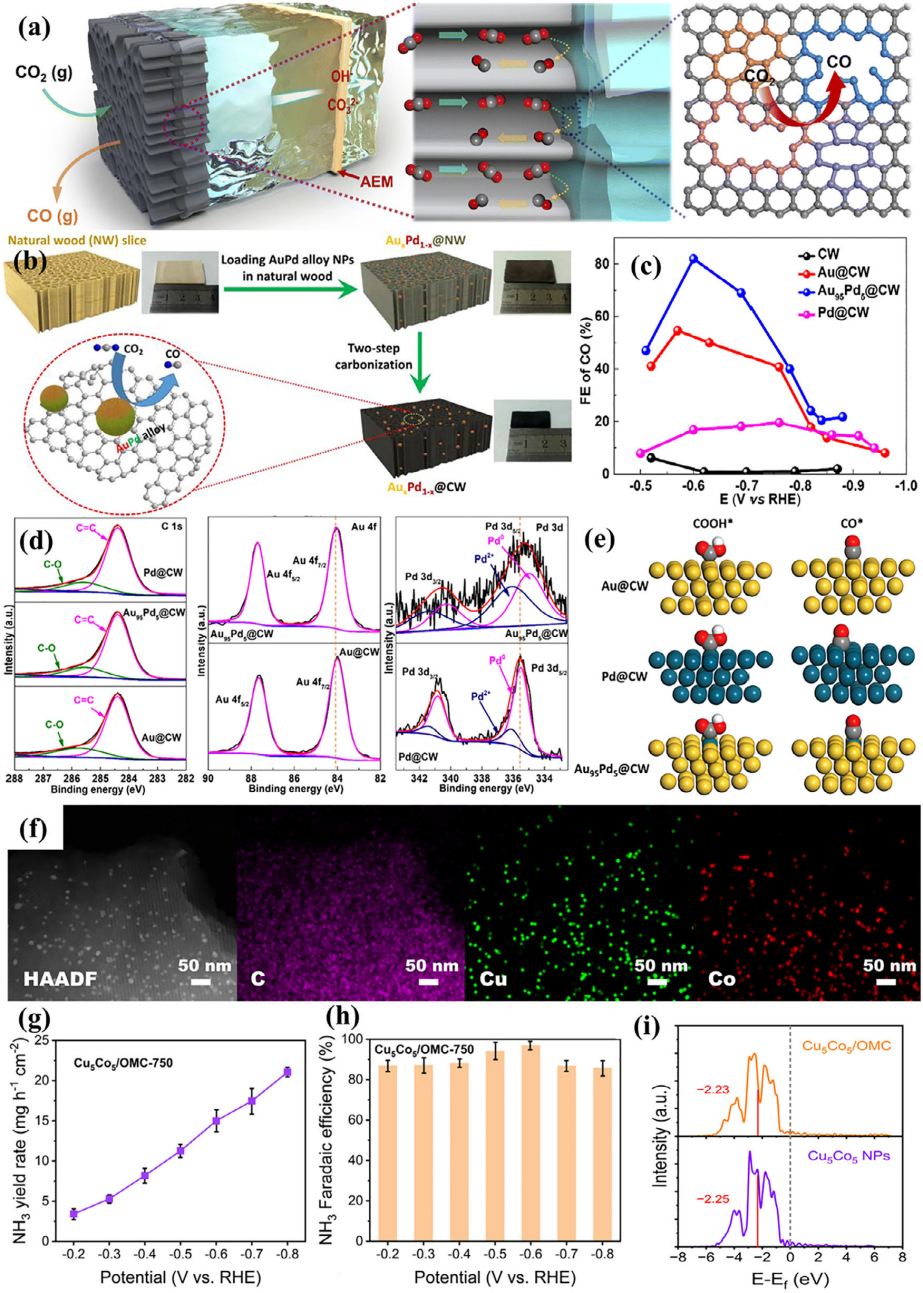
(a) Schematic illustration of the PTFE‐modified HDPCM‐based flow cell. Reproduced with permission [151]. Copyright 2024 Copyright Clearance Center. (b) Schematic illustration of the synthesis process of the Au*_x_* Pd_1−_ *_x_* @CW electrodes. (c) FE for CO generation on pristine CW, Au@CW, Au_95_Pd_5_@CW, and Pd@CW electrodes under varying applied potentials. (d) XPS survey spectra and high‐resolution XPS spectra of C 1s, Au 4f, and Pd 3d of Au@CW, Au95Pd5@CW, and Pd@CW electrodes. (e) DFT computational model for COOH* and CO* adsorption on Au@CW, Au_95_Pd_5_@CW, and Pd@CW. Reproduced with permission [80]. Copyright 2021 Elsevier Inc. (f) high‐angle annular darkfield scanning transmission electron microscopy (HAADF‐STEM) and elemental mapping of Cu_5_Co_5_/OMC. (g) NH_3_ yield rate, and (h) NH_3_ Faradaic efficiency of Cu_5_Co_5_/OMC from −0.2 to −0.8 V in 1 m KOH with 0.1 m KNO_3_. (i) PDOS and d‐band center of Cu_5_Co_5_/OMC and Cu_5_Co_5_ NPs. Reproduced with permission [152]. Copyright 2023, American Chemical Society.

The electrocatalytic conversion of CO_2_ into valuable chemical compounds and fuels through efficient reduction processes offers a viable approach to addressing the dual challenges of energy scarcity and environmental degradation [154, 155]. Nonetheless, the success of this methodology is intrinsically linked to the development of advanced electrocatalysts with superior performance characteristics [130]. In a significant contribution to this field, Min et al. [151] engineered carbon defects within self‐sustaining porous carbon film (HDPCM) electrodes, facilitating effective CO_2_RR in aqueous environments. This innovation marks a pivotal advancement in the realm of metal‐free carbon‐based electrocatalysts for CO_2_RR, as illustrated in Figure 8a. Building upon this foundation, the HDPCM electrode was subsequently transformed into a unified GDE through a straightforward hydrophobic modification, enabling its direct application in flow cell configurations to attain elevated current densities for CO_2_RR. Remarkably, a current density of −12.2 mA cm^−2^ and FE_CO_ of 79.2% were realized at a potential of −0.35 V relative to the RHE, representing a tenfold enhancement over the performance metrics observed in a conventional H‐cell.

The porous carbon structures derived from plant carbonization showed remarkable performance in CO_2_ reduction when loaded with metal catalysts in recent studies. Embedding metal nanoparticles directly into carbonized plants represents an alternative and promising strategy for enhancing electrocatalytic performance. Metallic and bimetallic nanoparticles are extensively utilized as electrocatalysts for CO_2_ reduction reactions (CO_2_RR) due to their catalytic properties [156, 157, 158, 159]. However, unsupported metal nanoparticles are prone to aggregation due to their high surface energy, which diminishes their catalytic activity during CO_2_RR [160]. To mitigate this challenge, nanoparticles are frequently modified with various supports to enhance dispersion and stability, achieved through strong interactions between the nanoparticles and their supports [161]. In a significant advancement in electrocatalytic CO_2_ reduction, Wang et al. [80] engineered AuPd alloy nanoparticles with adjustable compositions to create carbonized wood film‐based self‐supporting electrodes (AuPd@CW). These electrodes demonstrated exceptional performance, achieving a maximum FE_CO_ of 82% at a modest overpotential of 0.49 V, outperforming both Au@CW and Pd@CW counterparts (Figure 8b,c). XPS analysis revealed a pronounced synergistic interaction between Au and Pd within the Au*_x_* Pd_1−_ *_x_* alloy nanoparticles, facilitating efficient electron transfer between the two metals. This electronic modulation is crucial for optimizing the adsorption of reaction intermediates and desorption of final products, thereby enhancing the overall CO_2_RR efficiency (Figure 8d). Complementary density functional theory (DFT) simulations corroborated these findings, showing that the equilibrium adsorption energies of COOH* and CO* species on the AuPd nanoparticle surfaces favor CO* desorption, a key factor in improving electrocatalytic performance (Figure 8e).

In a parallel development, Tao et al. [150] introduced a novel GDE architecture utilizing carbonized wood as a dual‐function substrate—providing both electrical conductivity and porous structure—for reconstructed Ag nanoparticles (Ag@CW‐ER). This innovative design achieved a remarkable *j*_CO_ of −186.7 mA cm^−2^ at −1.31 V versus RHE, while maintaining a stable total current density of approximately −300 mA cm^−2^ over an 8‐h operational period. The uniform distribution of reconstructed Ag nanoparticles within the carbonized wood matrix created numerous active sites, significantly improving CO_2_ adsorption and activation during the electrochemical reduction process.

Notably, leveraging the hierarchical porous structure of the plants to construct porous nitrogen‐doped (N‐doped) carbon as a self‐supporting electrocatalyst effectively addresses the practical limitations of powdered carbon in CO_2_RR [162]. The hierarchical porous framework facilitates rapid mass transfer and provides an expansive surface area, thereby exposing more active sites. Furthermore, the incorporation of nitrogen species into the carbon matrix introduces potential active sites for CO_2_RR, promoting faster reaction kinetics and enhancing performance. Chang et al. have rationally designed and synthesized a self‐supporting single‐atom electrocatalyst for efficient CO_2_RR by anchoring highly active Ni‐N_4_ sites onto an N‐doped carbonized wood (Ni SAs‐NCW) matrix [149]. Similarly, Zhang et al. identified pyridine‐N doped onto carbonized wood as the most active site for selective CO_2_ reduction to CO. This configuration significantly lowers the free energy barrier for the formation of COOH* intermediates and enhances CO desorption efficiency [147]. Despite these advances, the limited diversity of products highlights significant opportunities for further exploration.

In addition, plant‐based electrodes, as a novel class of green electrocatalytic materials, exhibit distinct advantages and significant research potential in the electrocatalytic nitrate reduction reaction. This reaction typically produces multiple products, including nitrogen, ammonia, nitrite, and N_2_O, posing challenges in achieving both high selectivity and high activity simultaneously [163]. Enhancing reaction selectivity to favor the formation of a desired target product remains a formidable challenge, compounded by issues such as the catalyst's poor stability and high production costs [164]. Plant‐based electrodes address these challenges through surface modifications or doping, which allow for the regulation of their electrochemical properties [165]. For instance, nitrogen doping or oxide modifications can optimize the electron transfer pathways in plant electrodes, thereby modulating interactions between reactants and active sites to improve selectivity for specific products [153]. Charcoal electrodes derived from carbonized natural wood demonstrate exceptional performance, with a nitrate removal rate of 91.2%, selectivity of 98.5%, and an ammonium yield of 0.570 mmol L^−1^ h^−1^ cm^−2^ [46]. The higher sp^3^ carbon content and defect density in charcoal, relative to graphite, enhance electron transfer kinetics, thereby improving nitrate reduction sensitivity on the charcoal surface [46]. Furthermore, the natural porous structure of wood provides extensive distribution space for active sites, effectively enhancing the dispersion and catalytic activity of the material. Tian et al. [152] demonstrated that ordered mesoporous carbon (OMC) carriers facilitate the uniform dispersion of bimetallic CuCo active sites while promoting the effective enrichment of reactants (Figure 8f). Nitrate reduction was achieved with an ammonia yield of 21.05 mg h^−1^ cm^−2^ and a FE_NH3_ of 96.9% at −0.6 V versus RHE (Figure 8 g,h). This is attributed to the cooperation between OMC and CuCo bimetallic that obviously changes the electronic structure of Cu_5_Co_5_/OMC, thereby improving the adsorption and activation of reactants and intermediates (Figure 8i). This synergy enhances both the chemical activity and stability of metal catalysts. While plant‐based electrodes are still in the early stages of application in nitrate reduction reactions, their unique structural and functional properties underscore their significant potential as next‐generation electrocatalytic materials.

To sum up, noting that while the field is relatively new, functional treatments have led to promising results. For CO_2_ reduction, these electrodes provide a porous carbon framework that, when enhanced, demonstrates excellent performance. Key strategies include engineering carbon defects in metal‐free catalysts, loading the structure with metallic or bimetallic nanoparticles to achieve high selectivity for products such as CO, and incorporating nitrogen‐doping or single‐atom catalysts to create more active sites and improve reaction kinetics. In the area of nitrate reduction, plant‐based electrodes also show significant potential, addressing the key challenges of low product selectivity and catalyst instability. The performance can be improved through surface modifications like nitrogen doping or by using the plant‐derived carbon as a carrier for bimetallic active sites, which has been shown to achieve high ammonia yields and efficiency. Although applications in nitrate reduction are in the early stages, the unique properties of these materials position them as significant next‐generation electrocatalysts.

## 5 Conclusions, Challenges and Prospects

Plant‐based integrated electrodes represent a transformative approach to small molecule electrocatalysis, offering unique advantages in terms of sustainability, tunability, and cost‐effectiveness. By addressing challenges related to structural optimization, surface engineering, scalability, and mechanistic understanding, these materials can drive advancements in energy conversion, environmental remediation, and beyond. Through interdisciplinary research and innovative engineering, plant‐based electrodes can become a cornerstone of sustainable electrocatalysis, contributing to a more energy‐efficient and environmentally friendly future.

Despite their significant potential, plant‐based monolithic electrodes currently face several substantial hurdles that impede their widespread practical implementation. Performance‐wise, they struggle with inherently low conductivity and insufficient active sites, leading to high overpotentials and poor stability in reactions like water splitting and oxygen reduction. For CO_2_ and nitrogen reduction, issues such as low adsorption capacity, poor selectivity towards valuable products, and competing side reactions like HER limit their overall efficiency [166, 167]. Beyond catalytic performance, challenges extend to material properties and manufacturing; their natural heterogeneity results in inconsistent batch‐to‐batch quality, and they often exhibit poor electrical conductivity, low packing density, and inadequate mechanical stability. Furthermore, the lack of scalable and precisely controllable manufacturing processes remains a critical barrier to their broader adoption.

To fully realize the potential of plant‐based electrodes, a multi‐faceted approach focusing on strategic advancements is essential. Future efforts must prioritize optimizing their hierarchical porous structures through physical and chemical modifications to enhance ion transport and catalytic surface area [168]. Simultaneously, fine‐tuning surface properties is crucial for improving selectivity in complex reactions like CO_2_ reduction to valuable C_2+_ products. Besides, a deeper mechanistic understanding, guided by advanced characterization and computational modeling, will inform the design of next‐generation catalysts. Bridging the gap between academic research and industrial manufacturing is vital, necessitating the exploration of advanced fabrication techniques such as 3D printing for scalable and reproducible production. Future progress depends on advancing beyond laboratory metrics and developing scalable, cost‐effective manufacturing processes that preserve the material's unique porous architecture while aligning with green chemistry principles. Finally, to accelerate the lab‐to‐market translation of plant‐based electrode technologies, a strategic alliance between academia, industry, and government is essential [169]. This collaboration is necessary to bridge the gap between fundamental innovation, scalable manufacturing, and supportive policy, ultimately converting promising research into commercial green technologies.

## Author Contributions

**Ying Long**: literature review, investigation, data collection, analysis, drafting. **Zhijie Chen**: conceptualization, data analysis, methodology, writing – review and editing. **Jiangzhou Xie**: investigation, writing – review and editing. **Jinliang Zhu**: data interpretation, writing – review and editing. **Wei Wei**: data analysis, writing – review and editing, supervision; **Yi‐Ming Yan**: data analysis, writing – review and editing. **Bing‐Jie Ni**: supervision, methodology, project administration, supervision, writing – review and editing, finalization.

## Ethics Statement

Ethical approval was not required for this study as it did not involve human participants or animals.

## Conflicts of Interest

The authors declare no conflicts of interest.

## Data Availability

The data that support the findings of this study are available from the corresponding author upon reasonable request.
